# Current research into A20 mediation of allergic respiratory diseases and its potential usefulness as a therapeutic target

**DOI:** 10.3389/fimmu.2023.1166928

**Published:** 2023-03-28

**Authors:** Yan Liu, Kai Xu, Yin Yao, Zheng Liu

**Affiliations:** ^1^ Department of Otolaryngology-Head and Neck Surgery, Tongji Hospital, Tongji Medical College, Huazhong University of Science and Technology, Wuhan, China; ^2^ Hubei Clinical Research Center for Nasal Inflammatory Diseases, Wuhan, China; ^3^ Institute of Allergy and Clinical Immunology, Tongji Hospital, Tongji Medical College, Huazhong University of Science and Technology, Wuhan, China

**Keywords:** A20, allergic airway diseases, nuclear factor κB, protection, inflammation

## Abstract

Allergic airway diseases are characterized by excessive and prolonged type 2 immune responses to inhaled allergens. Nuclear factor κB (NF-κB) is a master regulator of the immune and inflammatory response, which has been implicated to play a prominent role in the pathogenesis of allergic airway diseases. The potent anti-inflammatory protein A20, termed tumor necrosis factor-α-inducible protein 3 (TNFAIP3), exerts its effects by inhibiting NF-κB signaling. The ubiquitin editing abilities of A20 have attracted much attention, resulting in its identification as a susceptibility gene in various autoimmune and inflammatory disorders. According to the results of genome-wide association studies, several *TNFAIP3* gene locus nucleotide polymorphisms have been correlated to allergic airway diseases. In addition, A20 has been found to play a pivotal role in immune regulation in childhood asthma, particularly in the protection against environmentally mediated allergic diseases. The protective effects of A20 against allergy were observed in conditional A20-knockout mice in which A20 was depleted in the lung epithelial cells, dendritic cells, or mast cells. Furthermore, A20 administration significantly decreased inflammatory responses in mouse models of allergic airway diseases. Here, we review emerging findings elucidating the cellular and molecular mechanisms by which A20 regulates inflammatory signaling in allergic airway diseases, as well as discuss its potential as a therapeutic target.

## 1 Introduction

The incidence rates of allergic airway disorders, including allergic asthma, allergic rhinitis (AR), and chronic rhinosinusitis (CRS), have increased dramatically in recent decades (1–4). Allergies affect the patients’ quality of life and can be life threatening in severe cases, resulting in a heavy financial burden on individuals and the society (5). Allergic airway diseases are characterized by prolonged and exaggerated type 2 immune responses to allergens and immunoglobulin E (IgE)-mediated hypersensitivity (6–8). Under normal physiological conditions, immune responses are tightly controlled at multiple levels to maintain immune homeostasis. Dysfunction of the immune network can lead to a hyperinflammatory state, resulting in the initiation and exacerbation of allergic diseases (8). The transcription factor nuclear factor kappa-B (NF-κB) is involved in both innate and adaptive immunity. Importantly, inappropriate NF-κB activation underlies allergic airway disorder development (8, 9). This phenomenon was confirmed in an allergic-asthma mouse model, where NF-κB translocation blockade led to decreased interleukin (IL)-4 and IL-17 production by type 2 helper T (Th2) and Th17 cells, respectively (10).

A20, encoded by the *TNFAIP3* gene, is a cytoplasmic ubiquitin (Ub)-modifying enzyme with dual ubiquitinating and deubiquitinating (DUB) activities (11). It acts as an endogenous negative regulator of NF-κB signaling and has anti-inflammatory and immunomodulatory effects in various inflammatory and autoimmune diseases (12). Single-nucleotide polymorphisms (SNPs) on the *TNFAIP3* locus are correlated to several inflammatory disorders, including rheumatoid arthritis (12, 13), systemic lupus erythematosus (14), and inflammatory bowel disease (15). A20 is involved in allergic disease pathogenesis (16). Reduced A20 expression is observed in the epithelium of asthmatic patients (17). Using *Tnfaip3* conditional knockout mouse models, its functions in lung in epithelial cells (16), dendritic cells (DCs) (18, 19), T cells (20), and mast cells (21) were explored. Moreover, novel anti-inflammatory functions of A20 have been identified, including those of inhibiting the activation of mitogen-activated protein kinases (MAPKs), modulating the activation of tumor necrosis factor (TNF) receptors and inflammasomes, and limiting the secretion of pro-inflammatory interleukins (22), demonstrating its potential role in anti-inflammation. Notably, intranasal administration of A20 has shown promising effects in alleviating allergic inflammation in animal models of allergic respiratory diseases (23, 24). Herein, we summarize the current data on the mechanism by which A20 regulates allergic diseases, its effects on allergic airway diseases on the basis of experimental evidence from human genetic studies and experimental evidence in animal models, and its potential as a therapeutic target.

## 2 Regulation of A20 and its relationship to NF-κB signaling in allergic disease

A20 is a highly conserved protein that comprising an amino-terminal ovarian tumor domain (OTU) at its N-terminus, and seven zinc finger (ZnF) domains at its C-terminus (11). The OTU domain is responsible for mediating deubiquitinating (DUB) activity, while ZnF4 and ZnF7 domains are responsible for K63- and M1-linked ubiquitination, respectively, thereby contributing to A20 ubiquitinating activity (12, 22).

Through altering the critical protein ubiquitination status in the toll-like receptor (TLR) and TNF receptor (TNFR) pathways, A20 is well-defined as a potent inhibitor of NF-κB signaling pathway (25, 26). Mechanistically, indicated proteins’ ubiquitination status depends on the OUT-domain’s DUB activity and the corresponding ZnF domains’ ubiquitinating activity of A20 (22). At least four out of seven ZnF domains at the C-terminus of A20 are involved in activating TNF-α-mediated NF-κB by recognizing and degrading receptor-interacting protein 1 (RIP1) (25, 27). Furthermore, A20 is induced by NF-κB activation in the TNFR signaling pathway, forming a negative feedback loop that causes the RIP1 inactivation through ubiquitination (25).

**Figure 1:**
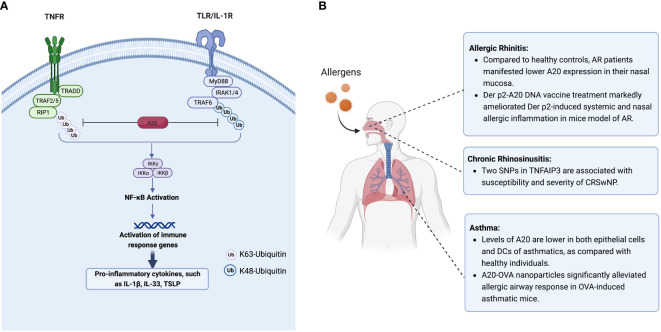
**(A)** A20 inhibits NF-κB signaling by targeting and degradation of key proteins in TNFR and TLR pathway. The canonical NF-κB pathway is triggered by signals from TNFR and TLR, leading to the activation of NF-κB, and further increased the transcription of cytokines IL-1β, IL-33 and TSLP. A20 inhibits the activation of IKK complex by degradation of ubquitinated RIP1 and TRAF6 in TNFR and TLR/IL-1R pathway, respectively. **(B)** The expression and regulation of A20 in allergic respiratory diseases. Figure panels were created with BioRender.com. NF-κB, Nuclear factor κB; TNFR, tumor necrosis factor receptor; TLR, toll-like receptor; TSLP, thymic stromal lymphopoietin; IL-1β/IL-33, interleukin 1β/33; IL-1R, interleukin-1 receptor; IKK, IκB kinase.

Recently, Das et al. summarized that several proteins regulating the expression and function of A20 proteins at different levels, including transcriptional, post-transcriptional, and post-translational levels (28). However, the upstream initiation and activation of A20 are not well defined. The function and activity of A20 appear to be associated with post-translational alternation, including phosphorylation and protein hydrolysis processes (**Figure 1A**). At the post-translational level, A20 is generally governed by inhibitory κB (IκB) kinases (IκK). A20 phosphorylation at serine 381 by an IκK leads to NF-κB pathway inhibition (11). In T and B cells, mucosa-associated lymphoid tissue transformation protein 1 (MALT1) mediates A20 cleavage following antigen receptor stimulation (29). The post-translational deubiquitylation modifications of A20 are related to K48- or K63-polyubiquitin chains (22). Multiple A20-binding proteins, including TNFR-associated factor 6 (TRAF6), Tax1-binding protein (TAX1BP1), and A20-binding nuclear inhibitor (ABIN), have been shown to regulate A20 activity (30). In the absence of TAX1BP1, the functions of A20 in regulating RIP1 binding, TRAF6 deubiquitylation, and further NF-κB activation suppression are impaired (30). ABIN-1 physically links A20 to IκK complex, facilitating A20-mediated de-ubiquitination of IκK component to inhibit NF-κB signaling (31).

## 3 A20 in allergic airway diseases

### 3.1 A20 in asthma

Allergic asthma is a common asthma type triggered by inhaled allergens and is defined by airway hyperresponsiveness, mucus hyperproduction, and eosinophilic inflammation (1). Inhaled allergens activate epithelial cells and DCs by pattern recognition receptors, leading to Th2 polarization and IgE production, ultimately resulting in the initiation of allergic inflammation (32). In this section, we summarize the phenotypes of existing asthma mouse models that specifically entail depletion of the *Tnfaip3* gene in different cell types as well as the critical *TNFAIP3* SNPs in asthmatic patients.

A20 protein is hardly detectable in most cell types under resting conditions except specific immune cells (peripheral T cells, DCs) and epithelial cells (18, 28). Studies have shown that A20 levels in both epithelial cells and DCs are significantly lower in asthmatic patients than those in healthy individuals (17). Roles of A20 in allergic asthma have been demonstrated by the observation that administration of A20 significantly attenuated allergic inflammation in asthmatic mice (23, 33). Using an adenovirus containing A20 cDNA, Kang et al. found that A20 significantly diminished inflammatory cell infiltration in the lungs and decreased IL-5 and IL-13 levels in the bronchoalveolar lavage fluid in ovalbumin (OVA)-induced asthmatic mice lungs, accompanied by compromised activation of RIP1 and NF-κB (33). In another study, Luo et al. encapsulated both A20 and OVA into poly(lactic-co-glycolic) acid (PLGA) to create a nano vaccine (23). They found that vaccinated mice showed significantly reduced serum OVA-specific IgE levels, alleviated local inflammatory cell infiltration, and increased regulatory T (Treg) cells numbers in the lungs (23), indicating that A20 might be a potential treatment target for allergic asthma (**Figure 1B**).

The airway epithelium is the first line of defense against microorganisms and allergens due to their interface location between the host and the environment. In the lung epithelium, A20 mediates protection against farm dust and endotoxin in patients with asthma (16). Compared with *Tnfaip3* wild type (*Tnfaip3* ^EC-WT^) controls, a lung epithelium *Tnfaip3* ***-*** knockout (*Tnfaip3* ^EC-KO^) mouse model produced less granulocyte-macrophage colony-stimulating factor and less eosinophil inflammation and Th2 response after house dust mites (HDMs) stimulation (16). Related phenomena were confirmed in bronchial epithelium air-liquid interface cultures from asthmatic patients (16). Lipopolysaccharide (LPS) low-dose exposure increased A20 expression in lung epithelial cells, significantly suppressed airway eosinophilia and bronchial hyperreactivity, and decreased allergen-specific IgE and IgG1 levels induced by HDMs in wild-type mice (16). These changes were not observed in the lung epithelial cells in the mice lacking A20 (16). Indeed, asthmatic patients had lower A20 levels in the lung epithelium than healthy subjects (16). Besides suppressing NF-κB activation, A20 also affected epithelial cells in other ways by facilitating staphylococcal enterotoxin B degradation in nasal epithelium *via* promoting endosomes and lysosome tethering (34), suggesting the central role of A20 in maintaining epithelial cell homeostasis.

DCs are antigen-presenting cells that regulate T-cell differentiation in the lung immune system. However, DCs activation depends on NF-κB activation. A20, an upstream regulator of NF-κB signaling pathway, is typically considered an anti-inflammatory mediator with therapeutic potential in certain allergic airway diseases (33). The expression of A20 in the DCs of the lungs has been correlated with Th2/Th17 cell differentiation in eosinophilic or neutrophilic asthma (18). Specific *Tnfaip3* deletion in mouse DCs leads to an increase in the levels of cytokines IL-6 and IL-23 correlated to the Th17 response, resulting in severe neutrophil inflammation (18). Conventional type 1 DCs (cDC1s) are a specific subset of DCs associated with antiviral and anti-tumor immune responses. Vroman et al. found that A20 knockout in cDC1s led to enhanced IL-12 production by cDC1s and increased PD-L1 expression in all pulmonary DC subsets (19). Mice with specific knockout A20 in the cDC1s displayed increased interferon-gamma (IFN-γ)-expressing CD8^+^ T cell numbers, and absence of Th2-driven eosinophilic airway inflammation in the lungs upon exposure to HDMs (19). These data indicate the importance of NF-κB signaling activation in the DCs for the differentiation of IFN-γ-expressing CD8^+^ T cells and Th17 cells. However, lower A20 expression in DCs in asthmatic patients than those in healthy controls was observed under unstimulated conditions (17). Therefore, careful consideration of animal models is required when assessing the role of A20 in DCs in asthma.

Increased Th2 cells are a hallmark of allergic asthma. A20, a negative regulator of NF-κB signaling, has been shown to inhibit the development of Th2-driven airway inflammation in OVA-challenged mice (33). Using mice with specific depletion of A20 in T cells (*Cd4* ^Cre^ *Tnfaip3* ^fl/fl^), Yokoyama et al. found that infiltration of eosinophils in the lungs, airway hyperresponsiveness, and levels of IL-5 and IL-13 in the lungs were significantly increased in the *Cd4* ^Cre^ *Tnfaip3* ^fl/fl^ mice compared to those in the wild type mice upon HDMs stimulation (20) (**Figure 1**). In *in vitro* studies, depletion of A20 in CD4^+^ T cells significantly enhanced IL-5 and IL-13 production under Th2 conditions. Mechanistically, the induction of GATA binding protein 3 (GATA3) was faster in CD4^+^ T cells from *Cd4* ^Cre^ *Tnfaip3* ^fl/fl^ mice than those from wild type mice, implying that A20 may act as a stabilizer of GATA3 levels during Th2 cell differentiation (20). Consistently, administration of an adenovirus containing A20 cDNA markedly reduced inflammatory cell infiltration in the lungs, inhibited inflammatory cytokines production in bronchoalveolar fluid, and prevented the development of airway hyperresponsiveness (33).

Using acute and chronic HDMs-driven asthma models, Vroman et al. recently found that conditional deletion of *Tnfaip3* gene in mouse myeloid cells promotes the secretion of Th17-inducing cytokines IL-1β, IL-6, and IL-23, thereby increasing Th17 cell numbers and exacerbating neutrophilic inflammation (18). In contrast, increased IL-6 and IL-12 robustly inhibited the differentiation of HDMs-specific Th2 cells (18).

**Figure 2:**
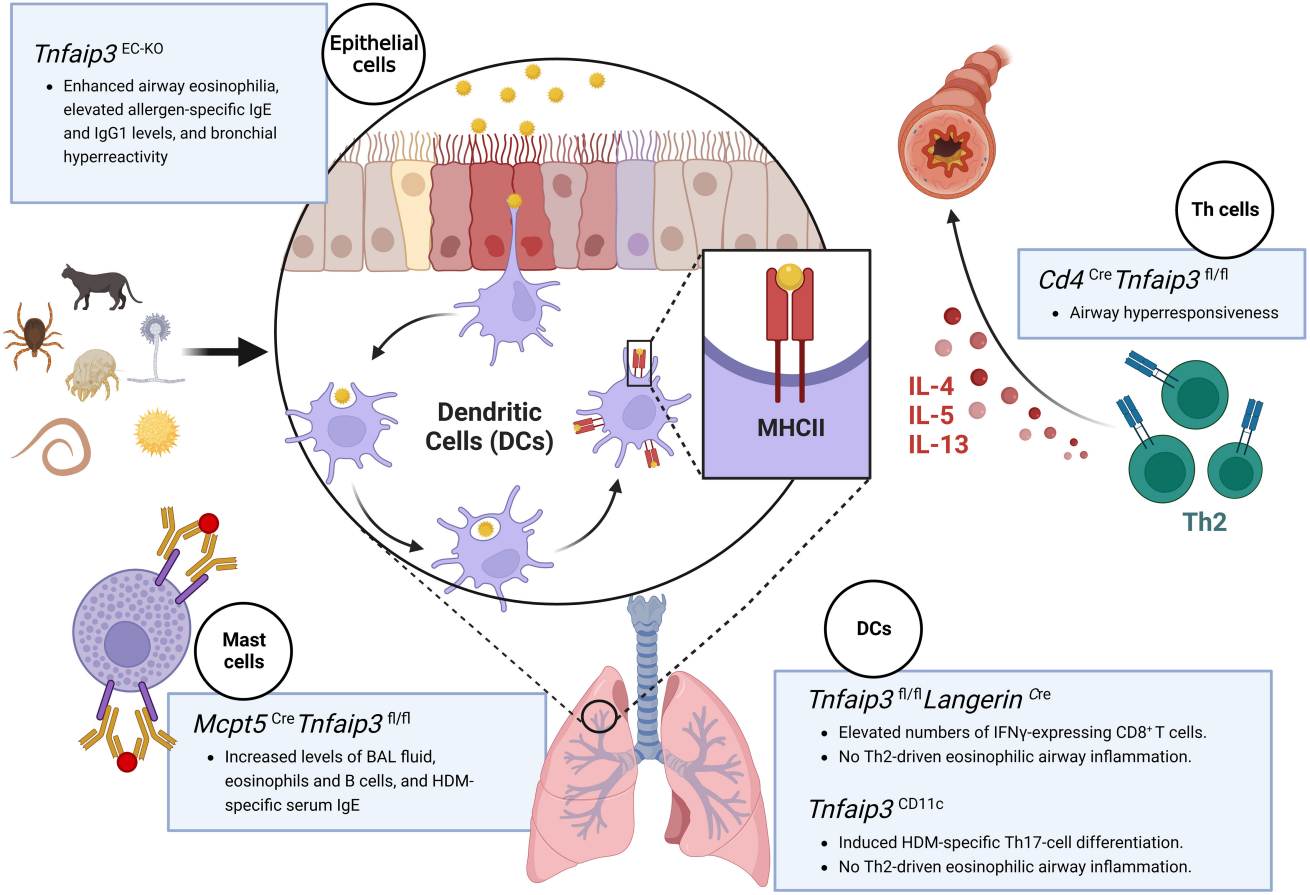
Schematic illustration of various cell-specific knockout mouse models used to study the role of the A20 protein in asthma. Asthma mouse models with specific knockout of the *Tnfaip3* gene in the epithelial cells (*Tnfaip3* ^EC-KO^) exhibit enhanced airway eosinophilia, elevated allergen-specific IgE and IgG1 levels, and bronchial hyperreactivity; mice with *Tnfaip3* gene knockout in the dendritic cells (*Tnfaip3* ^fl/fl^ Langerin^Cre^ or *Tnfaip3* ^CD11c^) exhibit the absence of Th2-driven eosinophilic airway inflammation, accomplished with elevated numbers of IFN-γ-expressing CD8^+^ T cells or increased levels of Th17 cell differentiation; mice with *Tnfaip3* gene knockout in T cells (*Cd4* ^Cre^ *Tnfaip3* ^fl/fl^) display airway hyperresponsiveness; and mice with *Tnfaip3* gene knockout in the mast cells (*Mcpt5* ^Cre^ *Tnfaip3* ^fl/fl^) display increased BAL fluid levels, serum IgE levels, and eosinophil and B-cell counts. Figure was created with BioRender.com. IgE/IgG1, immunoglobulin E/G1; IFN-γ, interferon-gamma; BAL, bronchoalveolar lavage.

Mast cell involvement in early- and late-phase asthma responses has been recognized. Using an A20 knockout mouse asthma model, Heger et al. reported that A20 does not govern the instant mast cell degranulation but acts as a negative feedback inhibitor of NF-κB pathway (21). They also found that loss of A20 in mast cells led to enhanced pro-inflammatory responses downstream of the mast cell antigen receptor module, TLRs, and IL-33R (21). Although mast cell-specific A20 ablation did not trigger any spontaneous pathology phenotype, it markedly elevated IL-33 production and aggravated allergic lung inflammation upon HDMs stimulation (21). A20 depletion, specifically in connective tissue-type mast cells (*Mcpt5* ^Cre^ *Tnfaip3* ^fl/fl^), prolonged the survival and enhanced the proliferation activity of LPS- and IL-33-activated mast cells, further promoting inflammation in an asthma model (21). To better illustrate the roles of A20 in asthma, we summarize the phenotypes associated with specific knockout of *Tnfaip3* in distinct cell types in mouse asthma models in **Figure 2**.

Genetic and environmental factors are involved in asthma pathogenesis, as illustrated by the significant difference in its prevalence among children living in urban versus rural areas (32). Genome-wide association studies of large patient cohorts indicated that the *TNFAIP3* gene should be considered as an asthma susceptibility locus (**Table 1**). In the GABRIELA study population, children growing up on farms had a higher risk of developing asthma than those growing up in urban areas, and this risk was associated with SNP rs2230926, a Phe127 to Cys127 mutation in exon 3 of TNFAIP3 (16). TNFAIP3 interacting protein 1 (TNIP1) interacts with A20 and suppresses the TNF-α-induced NF-κB activation. Li et al. found that several SNPs of the *TNIP1* gene, including rs1422673 and rs10036748, were associated with asthma risk (35). Taken together, these findings indicate that genetic factors of A20 play important roles in the development of asthma.

**Table 1:** Allergic airway diseases associated with TNFAIP3 single-nucleotide polymorphisms.

Linked diseases	SNP	Variation	Minor allele	Location	*P* value	OR (95%CI)	References
Allergic asthma	rs2230926	G/T	G	Exon 3	< 0.001	2.0 (1.4–3.0)	(16)
	rs1422673		T	Intron 5	< 0.001	0.63 (0.53–0.75)	(35)
	rs10036748	T/C	T	Intron1	< 0.001	0.68 (0.58–0.79)	(35)
Allergic rhinitis	rs9494885	C/T	C	Intron 3	< 0.001	1.94 (1.35–2.76)	(36)
	rs7753873	A/C	C	Intron 1	< 0.001	1.74 (1.26–2.40)	(36)
CRS	rs3757173	C/T	G	Intron 1	0.039	1.67	(37)
	rs5029938	C/T	T	Intron 2	0.019	1.95	(37)

SNP, single-nucleotide polymorphism; OR, odds ratio; 95% CI, 95% confidence interval; CRS, chronic rhinosinusitis

### 3.2 A20 in allergic rhinitis

AR, like asthma, is characterized by Th2-driven eosinophilic airway inflammation. Patients with AR had lower A20 expression levels in their nasal mucosa as compared with healthy controls (38). However, higher A20 expression was observed in nasal mucosa of OVA-induced AR mice compared to controls (38). Since A20 is an endogenous NF-κB inhibitor, its expression is strictly regulated (39). In an AR mouse model, antigen invasions, including OVA or pathogens, triggered pathogen recognition receptors, including TLRs, inducing A20 expression at both mRNA and protein levels (24). Subsequently, A20 plays a role in a negative feedback loop by inhibiting key pro-inflammatory signaling pathways, including those controlling NF-κB signaling (11, 22). Hu et al. reported that intranasal administration of an over-expression vector which could cause simultaneous overexpression of a dust mite antigen, *dermatophagoides pteronyssinus* (Der p) 2, along with A20 protein in a mouse AR model, led to significantly enhanced infiltration of mononuclear cells in the nasal mucosa, and attenuated the levels of Der p2−specific IgE, IL−4, and IL−13 in serum (24). CD4^+^CD25^+^Foxp3^+^ Treg populations were elevated substantially in both serum and spleen by upregulating A20 in Derp 2-induced AR mouse model (**Figure 1B**). It should be noted that there is a gap between experimental allergens animal models induced by certain allergens and allergic patients as the consequence of environmental exposures. The potential application of A20 for relieving the symptoms of AR patients warrants further exploration. The SNPs of *TNFAIP3* were also identified as susceptibility factors for AR. Ke et al. found that two SNPs of *TNFAIP3*, rs9494885 and rs7753873, were linked to AR’s susceptibility in the Chinese Han population (36) (**Table 1**).

### 3.3 A20 in CRS

CRS involves chronic inflammation of the mucous membranes of the nasal cavity and sinuses. Several studies have found associations between the *TNFAIP3* gene and the occurrence of CRS (37) (**Table 1**). Significantly, two SNPs (rs3757173 and rs5029938) in *TNFAIP3* are associated with the susceptibility and severity of CRS and nasal polyposis (37).

Activation of NF-κB signaling is associated with the production of various pro-inflammatory cytokines, including IL-1, IL-33 and thymic stromal lymphopoietin (TSLP), in human nasal epithelial cells (40). Glucocorticoid treatment, which is considered the first-line treatment of CRS (3), has been found to hinder the recurrence of CRS by inhibiting NF-κB signaling (41). Enhanced NF-κB pathway activation and increased IL-6 and IL-8 levels are observed in patients with CRS (42), implying the involvement of A20 in CRS pathogenesis and development. Nevertheless, neither expression levels nor the role of A20 in CRS has been investigated. Whether A20 contributes to the pathogenesis of CRS or nasal polyposis awaits to be explored. The application of an A20 overexpression vector in primary human nasal epithelial cells or conducting A20 depletion in cell-type specific CRS mice models would help illustrate the exact role of A20 in CRS, especially Th2-type CRS.

## 4 A20 as a potential therapeutic target in allergic airway diseases

A20 downregulation may be used as a biomarker to predict the development of childhood asthma (17). Moreover, induction of A20 expression after allergen-induced NF-κB activation is required to resolve inflammation (32, 43). Therefore, the pharmacological induction of A20 may provide resolutions for the inappropriate inflammatory immune response in allergic airway diseases. Farm dust and pathogen exposure, along with induction of A20 expression, reduced the childhood asthma risk (16). Using publicly available gene expression data and a statistically significant connections’ map, Malcomson et al. found that two medications, ikarugamycin and quercetin, significantly reduced A20 expression in both primary nasal and bronchial epithelium lines (44). Intranasally administration of IRL201104, a peptide derived from *M. tuberculosis* chaperonin 60.1, led to a significant increase in A20 expression in the lung of OVA-sensitized mice and a long-lasting anti-allergic effect, which at least partially explained the protective effects of tuberculosis against allergic diseases (45). A diterpenoid generated from plants called gibberellic acid has potent anti-inflammatory properties. Reihill et al. found that pre-treatment with gibberellic acid considerably increased A20 expression in LPS-induced bronchial epithelium and markedly decreased IL-6 and IL-8 production (46). Vitamin E in γ-tocotrienol natural forms upregulated A20 and inhibited NF-κB activation and its upstream regulator transforming growth factor β-activated kinase 1 in murine macrophages (47). Hand et al. observed that adiponectin, a pleiotropic adipokine, suppressed primary macrophage responses to LPS and pro-inflammatory fatty acids through the induction of A20 in adipose tissue (48). Chrysin is a flavone capable of exerting anti-neuroinflammatory effects in vegetables, fruits, plants, and honey, and it significantly enhanced A20 expression and inhibited LPS-induced NF-κB pathway activation and TRAF6 expression in primary microglial cells and cell lines (49).

Glucocorticoid administration is considered the most effective anti-inflammatory treatment for allergic airway diseases. A20 mRNA and protein expression levels were regulated by dexamethasone, a commonly used glucocorticoid, in human bronchial epithelial cell lines (50). Inflammatory cytokines also governed A20 expression (51). TNF-α and IL-1 promote A20 expression in various cell types. IL-4 and IL-13 significantly downregulate A20 expression in cultured sinonasal epithelial cells (51). Patients suffering from allergic illnesses, including asthma, CRS, and atopic dermatitis, have been demonstrated to benefit significantly from biologics that target IL-4 and IL-13, such as Dupilumab (52–59). Exploration of whether these biologics affect A20 expression in patients with allergic disorders is of great interest. Notably, A20 is expressed in various cell and tissue types. Mouse model studies have shown the effects of cell type-specific regulation of A20 expression, elucidating the A20 function in specific contexts. Therefore, pharmaceuticals targeting A20 to normalize allergic airway responses could be good candidates for future research on this topic.

## 5 Discussion

Allergic airway inflammation is usually characterized by airway hyperreactivity, increased mucus secretion, and eosinophil aggregation. Cytokines, including IL-25, IL-33, and TSLP are involved in activating the immune responses (1, 8). Accumulating evidence implies that A20 plays a protective role in allergic airway diseases. Findings from animal models reveal the A20 involvement in eosinophilic inflammation development; its role has been observed in the epithelium, T cells, DCs, and mast cells (16, 18–20). Conditional deletion of *Tnfaip3* gene in above mentioned cell types in mouse models of airway diseases resulted in an enhanced respiratory inflammatory response and increased local inflammatory factor release, thus facilitating our understanding of A20’s immunological and molecular mechanisms underlying allergic airway disorders. Multiple SNPs of A20/*TNFAIP3* are correlated to patients with allergic asthma, AR, and CRS (16, 35–37), supporting the notion that the genetic aspect of A20 is critical in allergic airway disorders. Despite the knowledge gained regarding the role of A20 in allergic airway diseases, several key questions still remain unsolved. Firstly, studies have demonstrated that A20 also protects cells from death, such as pyroptosis and apoptosis, independent of NF-κB signaling (11, 60). Thus, the additional roles of A20 in allergic diseases beyond orchestrating NF-κB signaling require further study. Secondly, other cell types, including follicular helper T cells, B cells, and group 2 innate lymphoid cells, are also crucial for the initiation and development of allergic diseases. Future cell-specific gene targeting studies related to the above-mentioned cell types may provide novel information on A20 function in allergic airway disorders. Furthermore, considering most of current researches are at preclinical level, clinical trials with large sample size are needed to evaluate the efficacy and safety of A20-targeted therapies in allergic respiratory diseases. Meticulously designed randomized cohort studies would help to select the patients who are likely to benefit from A20-targeted therapies. Lastly, the observation windows of current allergic airway disease mice models, either with OVA or HDMs stimulation, are usually shorter than two months, making it impossible fully manipulate the complex pathogenesis of allergic airway diseases in humans. Whether boosting A20 expression and/or its function is a promising strategy for resolving allergic airway disorders is yet to be elucidated.

## Author contributions

All authors contributed to the article and approved the submitted version.
